# Does discectomy improve low back pain as well as radiating pain in patients with lumbar herniated intervertebral disc (HIVD)?

**DOI:** 10.1097/MD.0000000000027559

**Published:** 2022-01-07

**Authors:** Sangbong Ko, Jaebum Kwon

**Affiliations:** Daegu Catholic University Medical Center, Daegu, Korea.

**Keywords:** discectomy, herniated disc, low back pain, lumbar spine

## Abstract

Most postoperative patients with herniated lumbar disc complained of lower leg radiating pain (LRP), referred buttock pain (RBP), and low back pain (LBP). When discectomy is performed, improvement in LRP is observed due to spinal nerve irritation. However, long-term LBP due to degenerative changes in the disc may occur postoperatively. In addition, limited research has been reported on the short-term (within 1 year) improvement in LBP after discectomy. This study aimed to evaluate the effectiveness of discectomy in reducing LBP within 1 year postoperatively.

Among the 183 patients who underwent discectomy performed by a single surgeon from January 2010 to December 2016, 106 who met the inclusion and exclusion criteria were enrolled. In the 106 patients who underwent lumbar discectomy, 3 types of spine-related pain were pre-operatively assessed and 3, 6, and 12 months postoperatively. Functional outcomes were evaluated, and quality of life was assessed 12 months postoperatively by using the Short-Form 36 questionnaire, which was subdivided into mental and physical components.

LBP showed both statistical and clinical improvement within the first 3 months postoperatively, but the improvement was not observed until 12 months postoperatively. RBP and LRP showed both statistical and clinical improvement within the first 3 months and further consistently showed statistical improvement. LBP improved clinically only until 3 months postoperatively regardless of the type of herniation.

LBP showed improvement within the first 3 months postoperatively and plateaued afterward, and RBP and radiculopathy showed consistent improvement until 12 months postoperatively. This may explain why patients from 12-month follow-up showed improvement in RBP and radiculopathy but not LBP.

## 1 Introduction

Patients who undergo surgical treatment for lumbar herniated intervertebral disc (HIVD) usually present with radiculopathy with low back pain (LBP) instead of radiculopathy alone. In such cases, LBP is referred to sciatica, but the main indication for surgery is radiculopathy instead of LBP.^[1]^ Although the symptom of most lumbar HIVD improves after conservative treatment, discectomy may be an option for the management of radiculopathy if conservative measures fail. Numerous studies have reported good postoperative results on discectomy as it is a big part in the field of spinal surgery.^[2–4]^ However, there is a limited research on the improvement in LBP after discectomy. Parker et al^[5]^ reported that although discectomy may be effective for the management of radiculopathy, the improvement in LBP is far less predictable and that LBP worsens after 1 to 2 years postoperatively. However, Toyone et al^[6]^ reported rapid improvement in LBP after discectomy, although the sample size of the study was small.

LBP can be caused by various spine-related disorders, including intervertebral disc (IVD) degeneration and disc herniation, but the underlying pathophysiological mechanisms have not been fully elucidated.^[7]^ During discectomy, aggressive excision of the IVD may reduce the recurrence risk of, but less favorable outcomes, such as degenerative IVD, may occur postoperatively. Minimal sequestrectomy may reduce some risk factors of LBP but increase the recurrence of lumbar HIVD.^[8]^ Several long-term studies have reported on degenerative changes in IVD and LBP after discectomy, but short-term studies (1 year after discectomy) have shown improvement in LBP and radiculopathy. Hence, a more comprehensive study must be conducted to further assess the inconsistent results of previous studies.

To evaluate the effectiveness of discectomy in reducing LBP within 1 year after surgery, we evaluated the serial improvements in the 3 types of pain that may be caused by the lumbar spine, namely LBP, referred buttock pain (RBP), and lower leg radiating pain (LRP), in patients who underwent discectomy.

## 2 Materials and methods

### 2.1 Patient selection and radiological evaluation

The participants eligible for the present study included patients diagnosed with single-level lumbar HIVD that is associated with symptoms of radiculopathy as confirmed on magnetic resonance imaging and those who have undergone discectomy due to radiculopathy that is unresponsive to 6 weeks of conservative treatment. The inclusion/exclusion criteria are summarized in Table 1. Among the 183 patients who underwent discectomy conducted by a single surgeon from January 2010 to December 2016, 106 agreed to undergo a postoperative follow-up for over a year.

**Table 1:** Inclusion and exclusion criteria.

Inclusion criteria	
1	Single level intervertebral disc herniation seen on MRI corresponding to radicular level
2	Primary radicular leg pain (below the knee for lower lumbar disc herniation, into the anterior thigh for upper lumbar disc herniation)
3	Evidence of nerve-root irritation with positive nerve-root tension sign (straight leg raise-positive between 30° and 70° or positive femoral tension sign)
4	Failure of at least 6 weeks of medical management, which included physical therapy, epidural injections, anti-inflammatory medications, and opioid analgesics.

Exclusion criteria	
1	Prior lumbar surgery
2	Cauda equine syndrome
3	Recurrent disc herniation
4	Presence of multilevel lumbar disc herniation or bilateral disc herniation
5	Rapid progressive severe motor deficit (less than grade 3 of 5)
6	Patients with secondary compensation (active medical or workmen's compensation lawsuit)
7	Vertebral fractures, spine infection or tumor, inflammatory spondyloarthropathy, pregnancy

IVD shown on the midsagittal T2-weighted fast spin-echo magnetic resonance imaging was evaluated pre-operatively according to the Pfirrmann classification.^[9]^ HIVD observed on the axial images was classified as subligamentous extrusion, transligamentous extrusion, and sequestrated type, according to the study by Fardon and Milette.^[10]^

All data of patients were reviewed retrospectively and there was no harm to the participants. For those reasons, the ethical approval was not necessary.

### 2.2 Study interventions

Standard open discectomy with examination of the affected nerve root was performed on the patients.^[4]^ After making a midline incision, paraspinal muscles were retracted to approach the interlaminar space. The medial border of the superior facet was excised in some patients for a better view during surgery. After approaching to the affected nerve root, the nerve roots were decompressed via unilateral partial laminectomy, and the herniated disc was excised. All surgeries were performed under microscopic guidance. After making small incisions in the annulus, disc fragments were excised, and curettage of the disc space was not carried out. Then, the free fragments were taken out from the disc space via normal saline irrigation alone. After microscopic examination of the canal, we probed the foramen for any residual discs or bony pathologies. Decompression of the nerve root enabled it to be freely mobile. Patients wore corsets and were encouraged to ambulate 2 days after surgery. Vigorous work or activities were restricted for 6 weeks. Patients were recommended to wear corsets until 2 to 4 weeks postoperatively to reduce trunk motion. After 6 weeks, the patients were wearing lumbosacral orthosis and underwent the same rehabilitation therapy for lower back.

### 2.3 Clinical outcomes (LBP and functional outcome) and quality of life measurements

The intensity of LBP, RBP, and LRP was recorded using the 10-mm visual analog scale (VAS), with a score of 0 indicating no pain and a score of 10 indicating the worst conceivable pain.^[11]^ Pain was assessed pre-operatively and 3, 6, and 12 months postoperatively. Thresholds for changes in LBP, LRP, or Oswestry Disability Index (ODI) were set using the minimum clinically important difference by Solberg et al.^[12]^ The cutoff values for the ODI, LBP, and LRP of the 2 groups were 20%, 2.5%, and 3.5%, respectively. With reference to such values, the cutoff value of RBP was 2.5.

Functional outcomes were evaluated 12 months after surgery using the ODI and the Rolland Morris Disability Questionnaire (RMDQ). Quality of life was assessed using the Short-Form 36 (SF-36) health survey questionnaire that was subdivided into mental and physical components. Survey using questionnaires and data collection were performed in person by an independent observer who was irrelevant to this study.

### 2.4 Statistical analysis

All analyses were performed with the Statistical Package for the Social Sciences software version 19.0. The epidemiological results were summarized using descriptive analysis and were presented as mean ± standard deviation for quantitative variables. Differences in the VAS scores and functional outcomes (ODI, RMDQ, SF-36 physical component score, and SF-36 mental component score) after surgery (initial vs 3 months, 3 months vs 6 months, and 6 months vs 12 months) were analyzed using paired *t* test, and the results were expressed as mean ± standard deviation. *P* values < .05 were considered statistically significant.

## 3 Results

### 3.1 Epidemiological results

The average age of the group comprising 106 patients was 49.24 ± 12.78 (range: 18–76) years. The average age of the male (n = 57) and female (n = 59) participants were 47.78 ± 12.46 (range: 22–76) and 50.86 ± 12.94 (range: 18–73) years, respectively. No significant differences were observed among the participants in terms of gender (*P* = .23). A total of 21 patients underwent surgery at L3–L4 level, 40 patients at L4–L5 level, and 45 patients at L5–S1 level. The average ODI and RMDQ were 13.09 ± 8.82 and 5.94 ± 6.43, respectively. The average SF-36 physical component score was 52.66 ± 25.62, and the average SF-36 mental component score was 57.15 ± 24.19. Total 106 patients were classified based on the degree of HIVD: subligamentous extrusion type (n = 31), transligamentous extrusion type (n = 34), and sequestration type (n = 41). Moreover, the patients were classified based on the degree of dis degeneration according to the Pfirrmann classification: grade 2 (n = 27), grade 3 (n = 57), grade 4 (n = 16), and grade 5 (n = 6) (Table 2).

**Table 2:** Epidemiological results.

		Number	Mean ± SD
Sex	Male	57	47.78 ± 12.46
	Female	49	50.86 ± 12.94
Functional outcome (postoperative 12 month)	ODI		13.09 ± 8.82
	RMDQ		5.94 ± 6.43
	SF-36 PCS		52.66 ± 25.62
	SF-36 MCS		57.15 ± 24.19
Level	L3–4	21	
	L4–5	40	
	L5–S1	45	
HIVD type	Subligamentous extrusion type	31	
	Transligamentous extrusion type	34	
	Sequestration type	41	
Pfirmann classification	Grade 2	27	
	Grade 3	57	
	Grade 4	16	
	Grade 5	6	

### 3.2 Time course and improvement of the 3 types of pain

The average LBP was 5.55 ± 2.747 pre-operatively, 2.15 ± 1.459 after 3 months, 2.21 ± 1.683 after 6 months, and 2.19 ± 1.928 after 12 months. The average RBP was 5.65 ± 2.564 after surgery, 2.51 ± 1.708 after 3 months, 1.60 ± 1.336 after 6 months, and 1.21 ± 1.343 after 12 months. The average LRP was 7.98 ± 1.179 after surgery, 2.01 ± 1.291 after 3 months, 1.17 ± 1.019 after 6 months, and 0.92 ± 1.131 after 12 months (Table 3).

**Table 3:** Time course and 3 types of pain.

VAS	LBP	RBP	LRP
Initial	5.55 ± 2.747	5.65 ± 2.564	7.98 ± 1.179
3 months	2.15 ± 1.459	2.51 ± 1.708	2.01 ± 1.291
6 months	2.21 ± 1.683	1.60 ± 1.336	1.17 ± 1.019
12 months	2.19 ± 1.928	1.21 ± 1.343	0.92 ± 1.131

LBP showed both statistical and clinical improvements within the first 3 months but was not maintained until after 12 months. RBP showed statistical improvement until after 3, 6, and 12 months and showed clinical improvement only until after 3 months. LRP showed significant improvement until after 3, 6, and 12 months but showed clinical improvement only until after 3 months. LBP had improved clinically (cutoff value > 2.5) and statistically (*P* value < .05) until the first 3 months after surgery, but it cannot be said that clinical improvement is statistically significant until 12 months after surgery. However, while RBP and LRP showed both statistical and clinical improvements during the first 3 months and consistently showed significant improvement, no clinical improvements were observed (Table 4).

**Table 4:** Time course and improvement of the 3 types of pain.

Time interval	3 types of pain	VAS	*P* value
Initial–3 months	LBP	3.396 (2.910–3.882)^†^	<.05^∗^
	RBP	3.142 (2.645–3.638)^†^	<.05^∗^
	LRP	5.972 (5.625–6.319)^†^	<.05^∗^
3 months–6 months	LBP	−0.057 (−0.365 to 0.252)	.717
	RBP	0.906 (0.637–1.175)	<.05^∗^
	LRP	0.840 (0.627–1.052)	<.05^∗^
6 months–12 months	LBP	0.019 (−0.259 to 0.296)	.893
	RBP	0.396 (0.132–0.660)	.004^∗^
	LRP	0.255 (0.050–0.459)	.015^∗^

### 3.3 Improvement in the 3 types of pain according to the type of HIVD

In patients with subligamentous extrusion type herniation, LBP improved within the first 3 months, but no improvement was observed after the 3 months. RBP improved until 12 months after surgery, and radiculopathy improved until 6 months postoperatively. Significant clinical improvement in all 3 types of pain was achieved only until 3 months postoperatively, but RBP and LRP respectively showed significant improvement until 12 and 6 months postoperatively.

In patients with transligamentous extrusion type herniation, LBP improved within the first 3 months and the last 6 months, and no improvement was observed at 3-month intervals. RBP improved within the first 6 months after surgery. However, the improvement was not significant within the last 6 months. LRP improved until 12 months postoperatively. Significant clinical improvement in all 3 types of pain was achieved only until after 3 months, but RBP and LRP respectively showed significant improvements until 6 and 12 months postoperatively.

In patients with sequestrated type herniation, LBP improved within the first 3 months but did not improve afterward. RBP and LRP improved only within the first 6 months postoperatively but did not significantly improve within the second 6 months. Significant clinical improvement in all 3 types of pain was achieved only until 3 months postoperatively, and RBP and LRP showed significant improvements until 6 months postoperatively (Table 5).

**Table 5:** Improvement of 3 types of pain according to type of HIVD.

Disc type	Time interval	3 types of pain	VAS	*P* value
Subligamentous extrusion type	Initial–3 months	LBP	3.806 (2.901–4.712)^†^	<.05^∗^
		RBP	4.000 (3.067–4.933)^†^	<.05^∗^
		LRP	6.290 (5.580–7.000)^†^	<.05^∗^
	3 months–6 months	LBP	0.194 (−0.346 to 0.733)	.469
		RBP	0.645 (0.176–1.114)	.009^∗^
		LRP	0.839 (0.483–1.194)	<.05^∗^
	6 months–12 months	LBP	−0.323 (−0.870 to 0.225)	.238
		RBP	0.645 (0.080–1.210)	.027^∗^
		LRP	0.065 (−0.379 to 0.508)	.768
Transligamentous extrusion type	Initial–3 months	LBP	2.676 (1.793–3.560)^†^	<.05^∗^
		RBP	2.824 (2.053–3.594)^†^	<.05^∗^
		LRP	6.029 (5.579–6.480)^†^	<.05^∗^
	3 months–6 months	LBP	−0.088 (−0.710 to 0.533)	.775
		RBP	1.088 (0.600–1.577)	<.05^∗^
		LRP	0.471 (0.170–0.771)	.003^∗^
	6 months–12 months	LBP	0.441 (0.037–0.846)	.034^∗^
		RBP	0.324 (−0.175 to 0.822)	.196
		LRP	0.559 (0.271–0.846)	<.05^∗^
Sequestrated type	Initials–3 months	LBP	3.683 (2.896–4.470)^†^	<.05^∗^
		RBP	2.756 (1.878–3.634)^†^	<.05^∗^
		LRP	5.683 (5.038–6.328)^†^	<.05^∗^
	3 months–6 months	LBP	−0.081 (−0.605 to 0.442)	.755
		RBP	1.054 (0.545–1.563)	<.05^∗^
		LRP	1.324 (0.038–2.328)	<.05^∗^
	6 months–12 months	LBP	−0.135 (−0.651 to 0.381)	.599
		RBP	0.297 (−0.118 to 0.712)	.155
		LRP	0.135 (−0.235 to 0.505)	.464

## 4 Discussion

It is extremely difficult to determine the cause of LBP.^[6]^ Kuslich et al^[13]^ reported that during surgery for HIVD under local anesthesia, stimulation of the affected nerve root induces leg pain but not back pain. Meanwhile, stimulation of the posterior part of the disc induces back pain alone. Furthermore, Nakamura et al^[14]^ and Ohtori et al^[15]^ reported that the posterior part of the disc is innervated by the sympathetic nervous system, which is the afferent nerve for discogenic LBP. In addition, sensory innervation of the posterior longitudinal ligament and lumbar dura mater has been reported.^[16,17]^ The occurrence of LBP in patients with lumbar HIVD can be explained by many mechanisms. Toyone et al^[6]^ reported that 40 consecutive patients with HIVD were treated with discectomy. All 40 patients were satisfied with the outcomes; hence, the authors suggested that nerve root compression caused by HIVD is a possible cause of LBP. In our study, patients showed significant improvement in LBP, RBP, and LRP 3 months postoperatively. The improvement in LBP in patients with lumbar HIVD after discectomy is still controversial based on studies with long-term follow-up period.^[2,5,18–20]^ In a trial including mean 38 months of follow-up, Hanley and Shapiro^[2]^ reported persistent LBP after discectomy in 14% of the patients. Moreover, Weber^[18]^ reported that in a 4-year follow-up trial, 11% of the patients presented with persistent LBP. Although the long-term degenerative changes and its consequential LBP may differ in patients depending on their individual conditions, factors that may affect LBP have been reported (although the exact criteria are not clear).^[19,20]^ However, they also reported that LBP persisted during the last follow-up compared with the pre-operative condition. Studies on the intensity of persistent LBP are limited. Parker et al^[5]^ reported about persistent LBP or radiculopathy in 3% to 34% of the patients within 6 to 24 months postoperatively and in 5% to 36% of the patients 24 months postoperatively. Moreover, increased LBP intensity without amelioration in ODI was observed in 3% of the patients after 3 months. Thus, LBP was similar or improved in 97% of the patients 3 months postoperatively, and changes in ODI were not observed. However, compared with the 3-month postoperative results, LBP and ODI worsened in 22% of the patients 12 months after surgery. However, as we did not evaluate the degree of pain improvement in our study, most patients showed improvement in LBP, RBP, and LRP 3 months after surgery. Ohtori et al^[20]^ also reported that the intensity of LBP continuously improved from the first year and until 2 years postoperatively compared with the pre-operative condition.

In a systemic literature review, Parker et al^[5]^ reported the recurrence of LBP, which improved after primary single-level discectomy in 3% to 34% of the patients 6 to 12 months after surgery and in 5% to 36% of the patients after 2 years. They insisted that this was irrelevant to recurrent herniation that occurred in 5.3% of the patients. Twenty-five percent of the patients experienced LBP and worsening of disability until 2 years postoperatively, but they insisted that LBP improved compared with the pre-operative condition. Carragee et al^[21]^ reported that 11% of the patients who have undergone discectomy presented with persistent LBP or radiculopathy, and 23% of the patients who have undergone aggressive discectomy had persistent LBP or radiculopathy. Moreover, the patients who have undergone limited discectomy presented with worse LBP compared with those who have undergone subtotal discectomy. The surgical spine surgeons may prefer to remove the herniated disc aggressively to reduce recurrence or remove the herniated disc less aggressively to reduce back pain caused by degenerative changes. However, consequential degenerative changes in IVD may still trigger LBP, and limited discectomy may cause recurrent herniation of the IVD.^[8]^

As there are no well-defined gold standard for the evaluation of therapeutic outcomes after the surgical management of lumbar HIVD, most clinicians evaluate postoperative outcomes by measuring the changes using ODI or the VAS.^[11]^ However, the correlation between the improvement of LBP and functional outcomes is unclear. Parker et al^[5]^ reported that recurrent or persistent LBP may deteriorate the functional outcomes, quality of life, and health utility of patients, but the reported results are based on a systemic review of several articles that may be biased due to various surgical techniques, broad entities of discectomy itself, and use of various evaluation methods. Therefore, we have serially compared the 3 types of pain caused by lumbar spine after minimizing the bias using a single surgical method and selected evaluation methods.

This study had several limitations. First, disc degeneration may be correlated with LBP, but we were not able to analyze such notion due to the small sample size. Therefore, further analysis of the factors, such as disc degeneration (Pfirmann classification) or end plate degeneration (Modic classification), must be conducted. Second, after discectomy, load application may increase in vertical motion in the facet joints and lead to overloading and development of LBP. In addition, although a time period of 3 to 6 months is assumed to be needed for LBP to develop in such manner, the amount of excised disc was not found to be correlated to the intensity of LBP. Third, only 103 patients who met the inclusion and exclusion criteria underwent follow-up for at least 1 year among 186 patients who were operated for 7 years were enrolled. There may be some limitations to the inclusion bias here.

## 5 Conclusion

Although all 3 types of pain that may be caused by the lumbar spine showed clinical improvement until 3 months postoperatively, no further improvement was achieved afterward. In other words, LBP improved within the first 3 months postoperatively and plateaued afterward, whereas LRP and RBP significantly improved within 12 months after surgery. RBP improved continuously in patients with subligamentous extrusion type, whereas LRP improved continuously in patients with transligamentous extrusion type. Patients with sequestrated type showed improvement in LRP and RBP within the first 6 months postoperatively and plateaued afterward.

## Author contributions

**Conceptualization:** Sangbong Ko.

**Data curation:** Sangbong Ko, Jaebum Kwon.

**Formal analysis:** Sangbong Ko.

**Investigation:** Sangbong Ko, Jaebum Kwon.

**Methodology:** Sangbong Ko.

**Project administration:** Jaebum Kwon.

**Writing – original draft:** Sangbong Ko, Jaebum Kwon.

**Writing – review & editing:** Sangbong Ko, Jaebum Kwon.
